# Mice lacking growth-associated protein 43 develop cardiac remodeling and hypertrophy

**DOI:** 10.1007/s00418-022-02089-x

**Published:** 2022-02-24

**Authors:** Michele Bevere, Caterina Morabito, Simone Guarnieri, Maria A. Mariggiò

**Affiliations:** 1 Department of Neuroscience, Imaging and Clinical Sciences, University “G. d’Annunzio” of Chieti-Pescara, 66100 Chieti, Italy; 2 Center for Advanced Studies and Technology (CAST), University “G. d’Annunzio” of Chieti-Pescara, 66100 Chieti, Italy

**Keywords:** GAP43, Cardiac hypertrophy, Calmodulin, Heart

## Abstract

Growth-associated protein 43 (GAP43) is found in skeletal muscle, localized near the calcium release units. In interaction with calmodulin (CaM), it indirectly modulates the activity of dihydropyridine and ryanodine Ca^2+^ channels. GAP43–CaM interaction plays a key role in intracellular Ca^2+^ homeostasis and, consequently, in skeletal muscle activity. The control of intracellular Ca^2+^ signaling is also an important functional requisite in cardiac physiology. The aim of this study is to define the impact of GAP43 on cardiac tissue at macroscopic and cellular levels, using GAP43 knockout (GAP43^−/−^) newborn C57/BL6 mice. Hearts from newborn GAP43^−/−^ mice were heavier than hearts from wild-type (WT) ones. In these GAP43^−/−^ hearts, histological section analyses revealed a thicker ventricular wall and interventricular septum with a reduced ventricular chamber area. In addition, increased collagen deposits between fibers and increased expression levels of myosin were observed in hearts from GAP43^−/−^ mice. Cardiac tropism and rhythm are controlled by multiple intrinsic and extrinsic factors, including cellular events such those linked to intracellular Ca^2+^ dynamics, in which GAP43 plays a role. Our data revealed that, in the absence of GAP43, there were cardiac morphological alterations and signs of hypertrophy, suggesting that GAP43 could play a role in the functional processes of the whole cardiac muscle. This paves the way for further studies investigating GAP43 involvement in signaling dynamics at the cellular level.

## Introduction

Growth-associated protein 43 (GAP43) is a highly conserved protein expressed in vertebrate species (Caprara et al. 2014). It is also known as neuromodulin because it was primarily isolated from synaptosomal membranes derived from rat brain, so for many years it was classified as a neuron-specific marker. Only later was GAP43 identified in other cell phenotypes, including glial cells and Schwann cells, especially in peripheral nerves undergoing degenerative processes (Curtis et al. 1992; Oestreicher et al. 1982).

In different species, GAP43 protein contains 194–238 amino acids, the sequence of which is characterized by three functional domains: the C-terminal domain, known as neuromodulin, is specific for GAP43; the N-terminal region, highly conserved in all vertebrates, contains 57 amino acids, whose first 10 ones are important for the protein binding to the plasma membrane; the domain near the N-terminal region is known as the IQ domain. This last domain consists of 12–15 amino acids and binds calmodulin (CaM), a protein that binds Ca^2+^ and therefore is involved in cellular processes modulated by intracellular Ca^2+^. The IQ domain of GAP43 begins its sequence with isoleucine and glycine binding before serine-41, the only site that can be phosphorylated by protein kinase C (PKC), and extends towards the C-terminal domain (Dent and Meiri 1998). In the nervous system, GAP43 has been defined as a “sponge” for CaM, being able to bind CaM, sequester it away from its downstream targets, and release it in response to changes in intracellular Ca^2+^ or to signals by PKC (Benowitz and Routtenberg 1997). In the latter signaling process, an important functional role is played by serine-41, which, when phosphorylated by PKC, decreases the affinity of GAP43 for CaM, allowing CaM release from GAP43-binding site. This accounts for GAP43’s role in regulating all CaM-mediated events that are triggered by changes in Ca^2+^ or after PKC activation (Dent and Meiri 1998). Although GAP43 has long been classified as a neuron-specific protein, there are studies that report the presence of this protein in non-nervous tissues as well. In particular, Stoker and collaborators in 1992 highlighted the presence of this protein in meromyosin-positive cells in the limbs of chicken embryo (Stocker et al. 1992). A few years later, in 1994, Heuss and collaborators revealed the expression of GAP43 in biopsy samples obtained from human skeletal muscle in subjects suffering from interstitial myositis, hypothesizing its involvement in the regenerative processes associated with muscle diseases (Heuss et al. 1994).

Data obtained in our laboratory, on the expression levels and intracellular localization of GAP43, showed that the protein expression follows a specific evolution related to the differentiation process from myoblasts to myotubes. Indeed, our experiments revealed that GAP43 was mainly localized within the nucleus in undifferentiated cells with diffuse patches within the cytoplasm, while in myotubes the protein localized at the cytoplasmic level, forming streaks transversal to the muscle fiber (Guarnieri et al. 2013).

Using adult skeletal muscle fibers derived from the extensor digitorum longus (EDL) muscle of the hind legs of C57BL/6 mice, it was possible to clarify in detail the position of GAP43 in relation to functional skeletal muscle fiber proteins, such as sarcomeric α-actinin and components of the calcium release units (CRUs). We demonstrated that GAP43 was positioned between CRUs and mitochondria, suggesting that GAP43 may play a key role in Ca^2+^ homeostasis also in skeletal muscle.

Adult GAP43^−/−^ mice have been shown to generate less force, and exhibit reduced body weight. Ultrastructural analyses of the diaphragm muscle (early maturing) and EDL (late maturing) from GAP43^−/−^ mice showed a delay in the degree of triads’ maturation as well as a reduced number of triad-junction complexes, despite the formation of the neuromuscular junctions being normal. These data highlight a possible role of GAP43 in those processes that accompany the development and functional maturation of skeletal muscle. Interestingly, Rahmati and Taherabad found lower levels of GAP43 in atrophied gastrocnemius muscle in diabetic animals, suggesting that the level of GAP43 could be a critical factor for skeletal muscle mass and size (Rahmati and Taherabadi 2021).

To elucidate the GAP43 functional role in skeletal muscle at the cellular level, intracellular Ca^2+^ homeostasis was investigated in myotubes deriving from differentiated satellite cells from wild-type (WT) and GAP43^−/−^ mice. Of note is the observation that GAP43^−/−^ myotubes showed profound alterations with Ca^2+^ release. Indeed, in GAP43^−/−^ samples, there was an increase in the number of myotubes with spontaneous oscillations having a higher frequency and amplitude than WT myotubes. Electrophysiological analyses showed that these alterations are due to increased L-type Ca^2+^ currents linked to an alteration of the CaM-operated control. It has been hypothesized that, due to its vicinity to the CRUs, GAP43, by interacting with CaM, provides a “functional microdomain” that locates the CaM near the CRUs. Thus, GAP43 indirectly modulates the activity of dihydropyridine receptor (DHPR) and ryanodine receptor (RyR) Ca^2+^ channels, thus influencing the dynamics of intracellular Ca^2+^ and the functional activity of myotubes (Caprara et al. 2016).

GAP43^−/−^ mice are bred by crossing heterozygous mice (GAP43^+/−^). GAP43^−/−^ newborns show decreased survival compared with GAP43^+/−^ and WT mice, which have a normal lifespan. In fact, about 45–60% of GAP43^−/−^ mice die within the first 48 h. About 45% die after 2 or 3 weeks after birth, at the time of weaning. Only a very small percentage of the knockout offspring survive past 3 weeks (about 5–10%) (Metz and Schwab 2004; Strittmatter et al. 1995). In this regard, it has been proposed that the presence of multiple aberrant patterns of synaptic connectivity could lead to deficits in feeding behavior or other autonomic functions, even if the sucking reflexes are present. Although this is a possible explanation, we wondered whether the elevated newborn mortality could be due also to alterations of vital organs, focusing our attention on the heart. The aim of this study was to investigate the main features of heart from GAP43^−/−^ mice to evaluate the presence of possible signs of dysfunctions. Our data highlighted that hearts from newborn GAP43^−/−^ mice showed hypertrophic features compared with hearts from WT ones. Histological section analyses revealed thicker ventricular wall and ventricular septum with reduced ventricular chamber area in GAP43^−/−^ hearts. Our results demonstrate that GAP43 protein could play an important role in the development of the cardiac muscle, though further studies are needed to investigate the intracellular pathways in which GAP43 is involved in cardiac muscle.

## Materials and methods

### Chemicals and materials

Unless otherwise indicated, cell culture media, sera, antibiotics, and cell culture dishware were obtained from Thermo Fisher Scientific (Monza, Italy), and reagents and standards from Merck Life Science (Milan, Italy).

### Animal care and use

C57BL/6 GAP43 heterozygous (GAP43^+/−^) mice were kindly provided by Dr Karina F. Meiri (State University of New York, USA) (Maier et al. 1999). The care and use of C57BL/6 GAP43^+/−^ mice to generate WT and GAP43^−/−^ mice strictly followed “*The Guiding Principles for the Care and Use of Animals*”, in accordance with the principles of the Declaration of Helsinki, with the European Community Council (86/609/CEE) and the Italian Government law on the protection of animals for experimental procedures in research laboratory (92/116). The experimentation and housing of the animals were conducted at the Center for Advanced Studies and Technology (CAST) with the consent of the “Organismo preposto al benessere animale” (OpBA) of the “G. d’Annunzio” University of Chieti-Pescara and with authorization no. 4739.n.ias of the Italian Ministry of Health [principal investigator (PI) S.G.].

### Mouse genotyping

The offspring of the GAP43^+/−^ mouse crosses were genotyped using a small fragment of the tail of neonatal mice, 24 h after birth. Experiments were performed using WT and GAP43^−/−^ mice. DNA extraction and amplification were performed using mice genotyping kits (KAPA Biosystems, Resnova S.r.l., Genzano di Roma, Italy), according to the manufacturer instructions. The DNA samples were amplified by PCR using the following primers: p1 (5′-GGCTCATAAGGC TGCAACCAAAAT-3′), p2 (5′-CCATCTCCCTCC TTCTTCTCCACA-3′), p3 (5′-CCGGCCGCTTGG GTGGAGAG-3′), and p4 (5′-TCGGCAGGAGCA AGGTGAGATGAC-3′).

### Cardiac mass index

Newborn mice were weighed and sacrificed according to the authorized procedure. Hearts were removed after performing midsection thoracotomy, rinsed in cooled phosphate buffer, and blotted dry; then the heart’s weight was measured. The cardiac mass index was calculated as ratio of heart weight to body weight. The values obtained were used to calculate the mean ± standard error of the mean (SEM).

### Heart isolation, fixation, and inclusion

WT and GAP43^−/−^ mice were sacrificed as described above, and after two washings in phosphate-buffered saline (PBS), isolated hearts were fixed in a solution of 10% formalin for 24 h. Fixed hearts were washed in PBS, appropriately oriented in the biocassettes, and treated using the EG-1160 embedding center and ASP 300 tissue processor (Leica Microsystems, Milan, Italy). Paraffin-embedded hearts were sectioned in slices of 6 μm at intervals of 150 μm from the cardiac base to the apex using RM2125 RT microtome (Leica Microsystems).

### Hematoxylin–eosin (H&E) staining

Sections from the medial region of each paraffin-embedded heart were stained with H&E using an automated TST 44 slide stainer (Medite Medical, Burgdorf, Germany) and mounted on glass slides. After H&E staining, cellular nuclei appeared stained blue, whereas the cytoplasm and extracellular matrix showed varying degrees of pink staining. The stained samples were observed using a straight transmitted light microscope Leica DMRD equipped with a 5×/0.8 numerical aperture (NA) objective (Leica Microsystems). Images were acquired using a DFC550 camera (Leica Microsystems) and analyzed with the software of Leica LAS (Leica Application Suite, Leica Microsystems) to perform quantitative measurements. For each section, the wall thickness of the left ventricle was measured at three different points (anterior, medial, and posterior). The values obtained were used to calculate the mean ± SEM.

### Masson’s trichrome staining

Masson’s trichrome staining was used to distinguish collagen from cardiac muscle tissue. Paraffin-embedded heart sections, obtained as reported above, were incubated for 10 min with iron hematoxylin solution, washed in distilled water, and processed with picric acid alcoholic solution for 1 min. Then, samples were washed in distilled water and treated with Ponceau acid fuchsin for 1 min. After washes, samples were fixed with phosphomolybdic acid 1% for 5 min, stained with aniline blue for 1 min, and dehydrated in ethanol and xylene. The collagen appeared stained blue, nuclei black, myocardium red, and erythrocytes yellow. The stained samples were observed using a straight transmitted light microscope Leica DMRD (40×/1.0 NA objective, Leica Microsystems). The images were acquired using a Leica camera (model DFC550, Leica Microsystems) and analyzed with the software of MetaMorph 6.1 (Molecular Devices, Downingtown, PA, USA). The area of collagen and the area of cardiac muscle are expressed as percentage ratio to total area, and data are reported as mean ± SEM.

### Immunofluorescence analyses

Papillary muscles were dissected from hearts of adult (3 months) WT mice and fixed with 4% paraformaldehyde for 10 min at room temperature. After three washes in PBS, the samples were permeabilized with a 0.2% Triton X-100 solution for 10 min, incubated in blocking buffer (PBS containing 10% goat serum) for 1 h at room temperature, then incubated overnight at 4 °C with primary antibodies anti-GAP43 (rabbit polyclonal anti-GAP43, HPA-GAP43, dilution 1:500, Merck Life Science) and mouse monoclonal anti-α-actinin 2 (1:1000 dilution, Merck Life Science). The primary antibody anti-GAP43 (HPA-GAP43, Merck Life Science) was validated by western blot using muscle GAP43 knockout extracts as negative control and brain tissue homogenates from WT mice as positive control (Caprara et al. 2016). Primary antibodies were revealed by 1 h incubation with secondary goat anti-rabbit IgG Alexa 488 (dilution 1:200, Thermo Fisher Scientific) together with goat anti-mouse IgG Alexa 543 (dilution 1:200, Thermo Fisher Scientific). The nonspecific binding of the secondary antibody was tested by performing the staining protocol by omitting the primary antibody. Images were acquired using a Zeiss LSM510 META system (Carl Zeiss, Jena, Germany) equipped with a Zeiss Axiovert 200 inverted microscope, a Plan Neofluar oil-immersion objective (100×/1.3 NA), and LSM 3.0 image analysis software (Zeiss).

### Cross-sectional area

Paraffin-embedded heart sections, obtained as reported above, were stained with wheat germ agglutinin conjugated with tetramethyl rhodamine (WGA-TRITC) diluted 1:100 in PBS for 30 min at room temperature. After washing with PBS, sections were treated with antifade mounting medium (Slow-fade, Thermo Fisher Scientific). The samples were observed using a Zeiss LSM800 URGB confocal microscope equipped with an upright AxioObserver Z1 microscope and a 40×/1.3 NA oil immersion objective and ZEN Blue 2.1 software (Zeiss). For each section, at least 40 cardiac fibers in three different fields were acquired. Acquired images were analyzed with ImageJ software (Fiji distribution, NIH Image). Fiber area was calculated by tracing the WGA-TRITC fluorescence signal. Data are reported as mean ± SEM.

### Western blotting

Protein extracts for western blotting were isolated from brain and heart of the mice. The tissues were homogenized with a Kontes glass potter (DWK Life Sciences, Mainz, Germany) in ice-cold RIPA lysis buffer (R89901, Thermo Fisher Scientific Inc). After centrifugation (10,000*g*, for 10 min at 4 °C), the protein concentrations in the supernatants were determined (Bio-Rad protein assay; Bio-Rad Laboratories, Segrate, Italy). Samples (20 μg) were resuspended in Laemmli buffer and separated by SDS-PAGE on 8%, (w/v) homogeneous slab gels, and then electroblotted onto nitrocellulose membranes (Amersham Protran 0.45 µm NC, GE Healthcare, Milan, Italy). Equal loading of the protein samples and transfer efficiency were monitored using Red Ponceau S (Merck Life Science) staining of each membrane, and Coomassie blue staining (0.25% Coomassie blue solution; R 250/G 250 1:1; Bio-Rad) of the gels. The membranes were blocked with a TBS-Tween 0.1% solution with milk 5%, and then incubated with the following primary antibodies: HPA-GAP43 rabbit polyclonal anti-GAP43 (1:1000 dilution, Merck Life Science); mouse monoclonal antibody anti-β-myosin heavy chain (MYH) (1:1000 dilution; Santa Cruz Biotechnology, Santa Cruz, USA); mouse monoclonal antibody anti-MYH7 (1:1000 dilution; Santa Cruz Biotechnology). The membranes were then incubated with horseradish-peroxidase-conjugated sheep anti-IgG, with the relevant proteins detected using chemiluminescence kits (1:10,000 dilution, GE Healthcare) and an image acquisition system (Uvitec, Cambridge, UK). A mouse monoclonal anti-GAPDH antibody (1:10,000 dilution; Merck Life Science) was used as the loading control.

### Statistical analyses

Data are expressed as the mean ± standard error of the mean (SEM) and compared using Student’s *t*-test with Prism5 software (GraphPad, San Diego, CA, USA). *p*-Values < 0.05 were considered statistically significant.

## Results

### GAP43 expression and localization in cardiac muscle

**Fig. 1:**
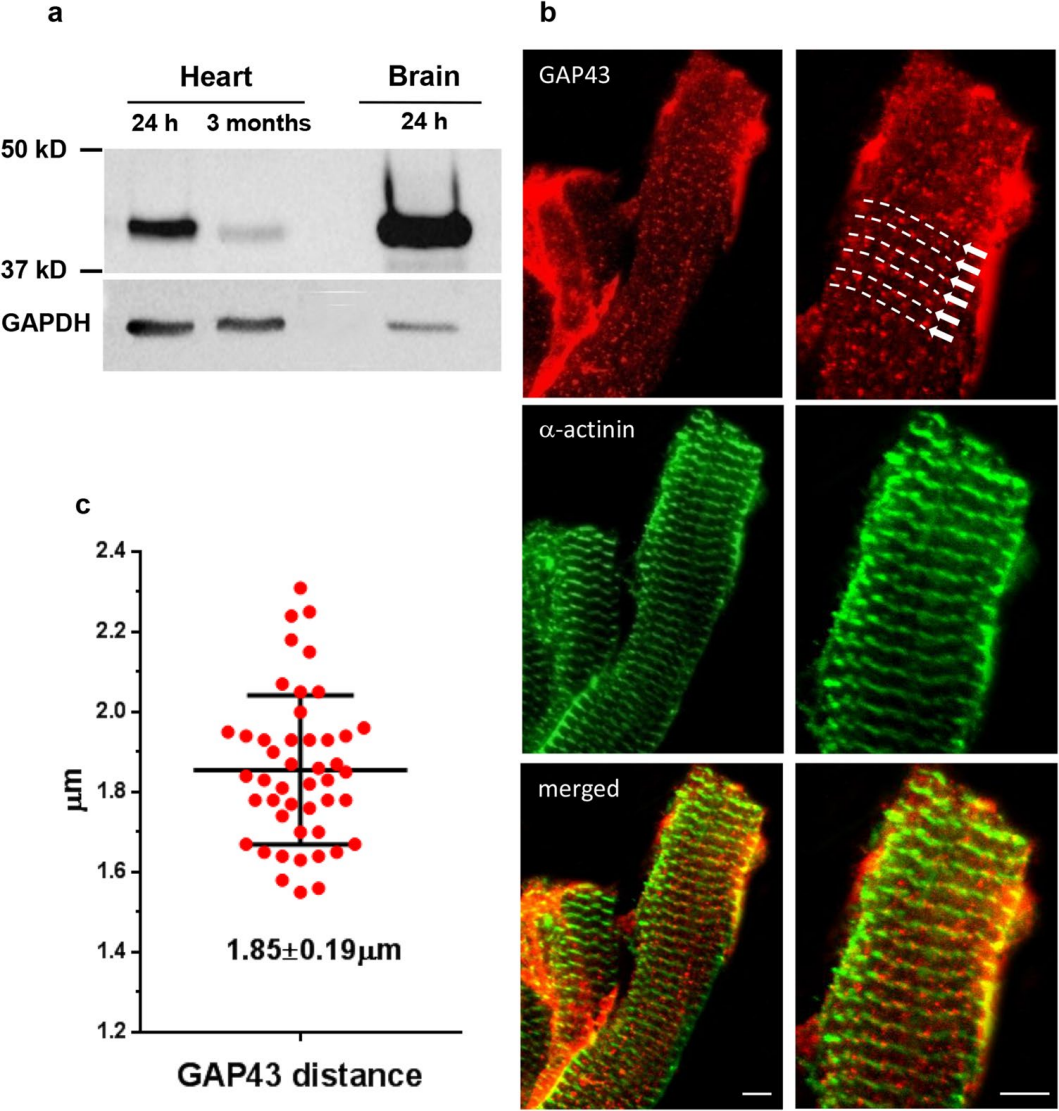
GAP43 expression and localization in the heart. **a** Representative western blots of GAP43 expression in hearts from WT newborn mice at 24 h after birth (heart 24 h) or from WT adult mice (heart 3 months), and in brain from WT mice at 24 h after birth (brain 24 h). Total proteins were loaded onto SDS-PAGE at 40 μg for heart homogenates and 2 μg for brain. The same membrane was detected also for anti-GAPDH antibody as loading control. **b** Representative images of adult papillary muscle fibers immunostained to detect GAP43 and α-actinin localization. The arrows and dashed lines indicate immunofluorescence for GAP-43, which reveals a dotted pattern depicting lines across the length of the fiber. Scale bar, 5 µm. **c** Distances between GAP43-positive spots. Data are expressed as mean ± SEM

Heart muscle homogenates were obtained from WT C57BL/6 mice at 24 h after birth and in adult mice (3 months), and used to define the expression levels of the GAP43 in the heart. Heart protein samples and homogenates from WT mice brain (representing a positive control for GAP43 immunodetection) were separated by electrophoresis in SDS-PAGE, and subjected to immunoblotting. In the samples obtained from the heart tissue (at 24 h or at 3 months of age), there was a specific band at the molecular weight characteristic of GAP43 (~ 43KDa) as well as in the brain sample used as positive control (see Fig. 1a). Comparing the two heart samples, it is interesting to note that the protein had different levels of expression in relation to mouse age and, consequently, heart development. In particular, it was observed that in newborns (heart 24 h, Fig. 1a) the levels of GAP43 in the neonatal heart tissue were higher than those found in the heart of adult animals (heart 3 months, Fig. 1a).

To analyze the localization of the GAP43 in the heart muscle, we used confocal microscopy and dissociated fibers derived from heart papillary muscles obtained from WT C57BL/6 adult mice. The localization of GAP43 was compared with the labeling of the α-actinin whose localization is known at the level of the Z line. The immunofluorescence signal for GAP43 revealed a dotted pattern that drew transverse lines (Fig. 1b). The measure of the mean distance (± SEM) between the spots was found to be 1.85 ± 0.19 µm (Fig. 1c). Furthermore, GAP43 appeared to localize close to α-actinin, but not co-localizing, and the GAP43 spots appeared to decorate the two sides of the Z-line (Fig. 1c).

### GAP43 knockout mice have greater cardiac mass index

**Fig. 2:**
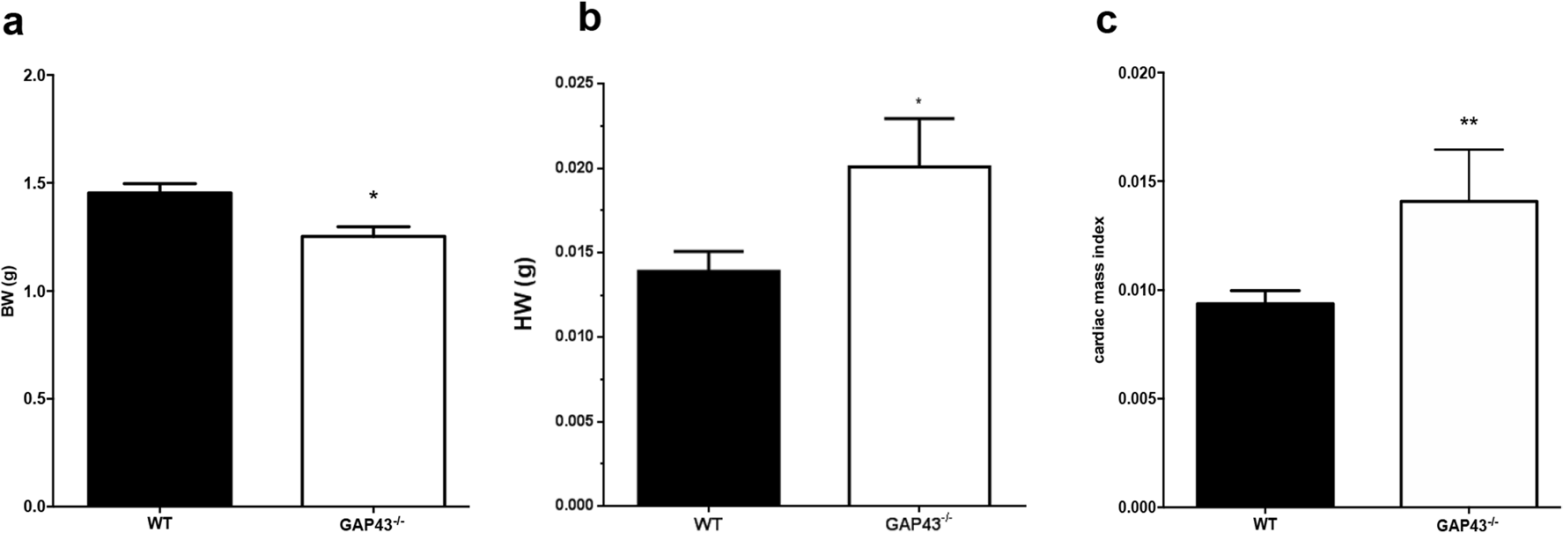
Cardiac mass index in WT and GAP43^−/−^ mice. **a** Comparison of body weights (BW) between WT (1.453 ± 0.043 g, *N* = 49) and GAP43^−/−^ (1.342 ± 0.062 g, *N* = 18) newborns. **b** Heart weights (HW) of WT (0.014 ± 0.001 g, *N* = 49) and GAP43^−/−^ (0.020 ± 0.003 g, *N* = 18) newborns. **c** Cardiac mass index was calculated as the ratio between the heart weight and the body weight of the same WT or GAP43^−/−^ newborn mouse. All data are expressed as mean ± SEM. ^∗^*p* < 0.05 versus WT; ^∗∗^*p* < 0.01 versus WT

At 24 h after birth, the GAP43^−/−^ mice appeared very similar to the WT littermates, but their body weights were slightly, but significantly, lower than those of WT (1.453 ± 0.044 g, *N* = 49 versus 1.251 ± 0.045 g, *N* = 15, respectively, *p* = 0.0181, Fig. 2a). Nevertheless, the hearts of GAP43 homozygous knockout mice weighed more than those of WT (0.020 ± 0.003 g, *N* = 18 versus 0.014 ± 0.001 g, *N* = 49, respectively, *p* = 0.0203; Fig. 2b). One-day-old homozygous knockout mice exhibited a ratio of heart weight to body weight about 1.5-fold that of WT mice (0.015 ± 0.002 *N* = 25 versus 0.009 ± 0.001 *N* = 61, respectively, *p* = 0.0021, Fig. 2c). The same analysis was performed for lung-to-body weight and liver-to-body weight ratios for the two genotypes; these ratios showed no detectable differences between homozygous knockout and WT mice (data not shown).

### Morphometric analyses

**Fig. 3:**
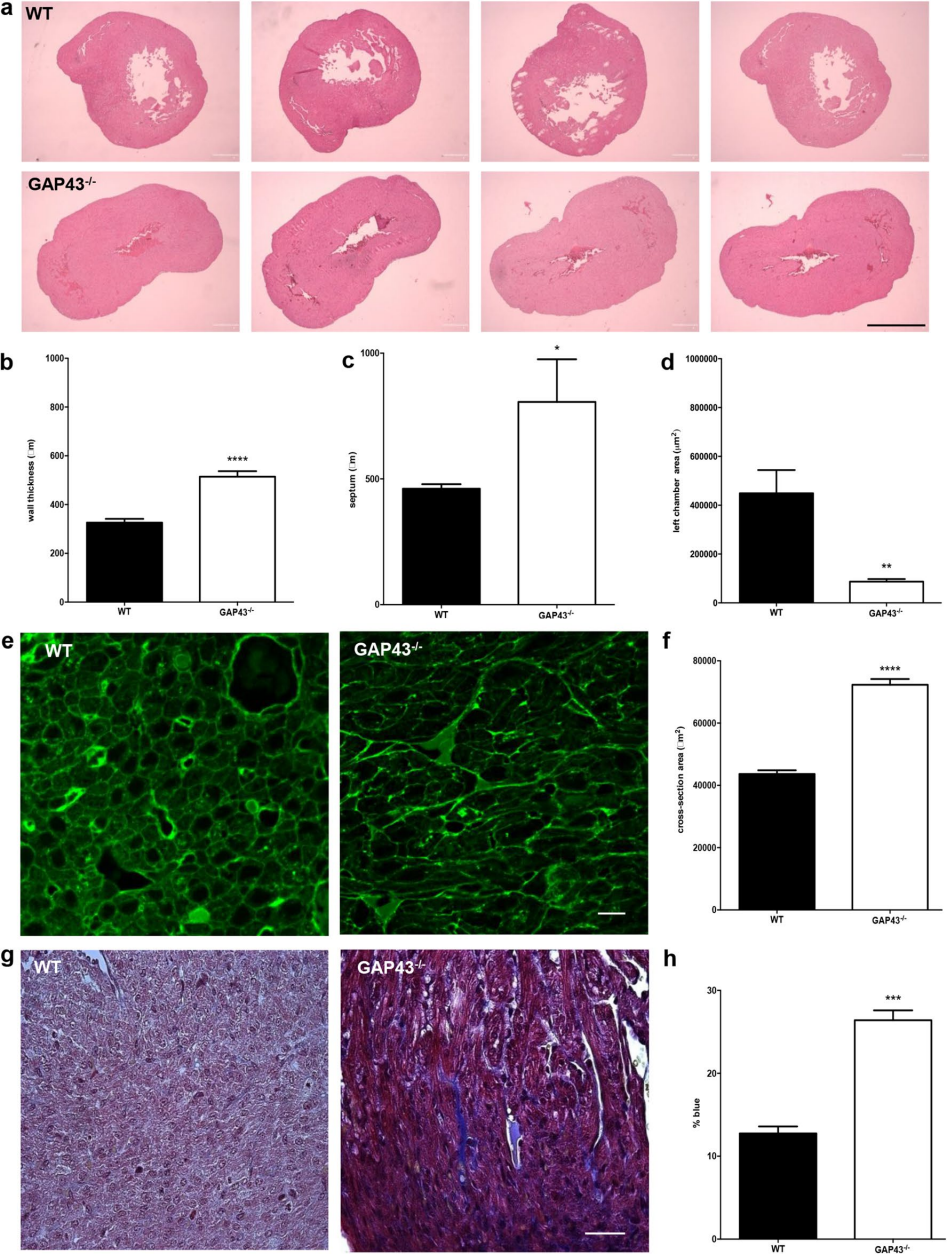
Morphometric analyses in hearts from WT and GAP43^−/−^ mice. **a** Representative images of transversal sections of WT and GAP43^−/−^ hearts stained with hematoxylin–eosin (scale bar, 1 mm). **b** Measurement of left wall thickness. **c** Measurement of septum thickness. **d** Measurement of left chamber free area. **e** Representative images of confocal acquisition of cross-sections obtained from the left ventricular wall and stained with wheat germ agglutinin conjugated with tetramethyl rhodamine and pseudocolored in green (scale bar, 10 µm). **f** Quantitative analyses of cross-sectional area, tracing the fluorescent WGA-TRITC signals (for each section, at least 40 cardiac fibers were measured). **g** Representative images of WT and GAP43^−/−^ heart sections stained with Masson’s trichrome (scale bar, 20 µm). **h** Quantitative analysis of blue staining percentage, which indicates the percentage of collagen in WT and GAP43^−/−^ hearts. For panels **b**, **c**, **d**, **f**, and **h**, data are expressed as the mean ± SEM from three independent experiments. ^∗^*p* < 0.05 versus WT; ^∗∗^*p* < 0.01 versus WT; ^∗∗∗^*p* < 0.001 versus WT

Transversal sections from heart medial region were obtained from newborn (24 h) WT and GAP43^−/−^ mice and processed for hematoxylin–eosin staining (Fig. 3a). Using the images acquired from each section, the wall thickness of the left ventricle at three different points (anterior, medial, posterior), the septum thickness, and the left chamber area were calculated for each sample; the values were used to calculate the mean values (Fig. 3b). Statistical analysis showed that left wall thickness was about 1.5-fold higher in samples from GAP43^−/−^ mice compared with WT hearts (514 ± 22 versus 326 ± 15 µm, respectively, *p* < 0.0001) (Fig. 3b). A similar relation was observed for septum thickness (806 ± 170 versus 461 ± 18 µm, GAP43^−/−^ versus WT, *p* = 0.0236) (Fig. 3c), while the free area of ventricular chamber was about fivefold lower than in WT (448.971 ± 949.99 versus 868.22 ± 106.95 µm^2^, GAP43^−/−^ versus WT, *p* = 0.0029) (Fig. 3d). For further morphometric analysis of the hearts, cross-sectional area was analyzed. For this purpose, the cross-sections of the hearts were stained with WGA to depict the sarcolemma. Figure 3e shows representative images of the acquired microscopic fields. The WT samples showed a regular honeycomb pattern with fibers of similar diameter, while in the cross-sections of GAP43^−/−^ samples the organization of fibers appeared to be disarrayed, with fibers of increased diameter. Quantifying the cross-sectional area of the fibers, we observed an increased mean value in GAP43^−/−^ samples versus WT ones (72.3 ± 18.2 µm^2^ *n* = 229 versus 43.6 ± 11.8 µm^2^ *n* = 229, *N* = 3, respectively, Fig. 3f). Interestingly, Masson’s trichrome staining highlighted in GAP43^−/−^ samples an intense presence of blue staining due to collagen deposit between fibers (Fig. 3h).

### Myosin expression levels

**Fig. 4:**
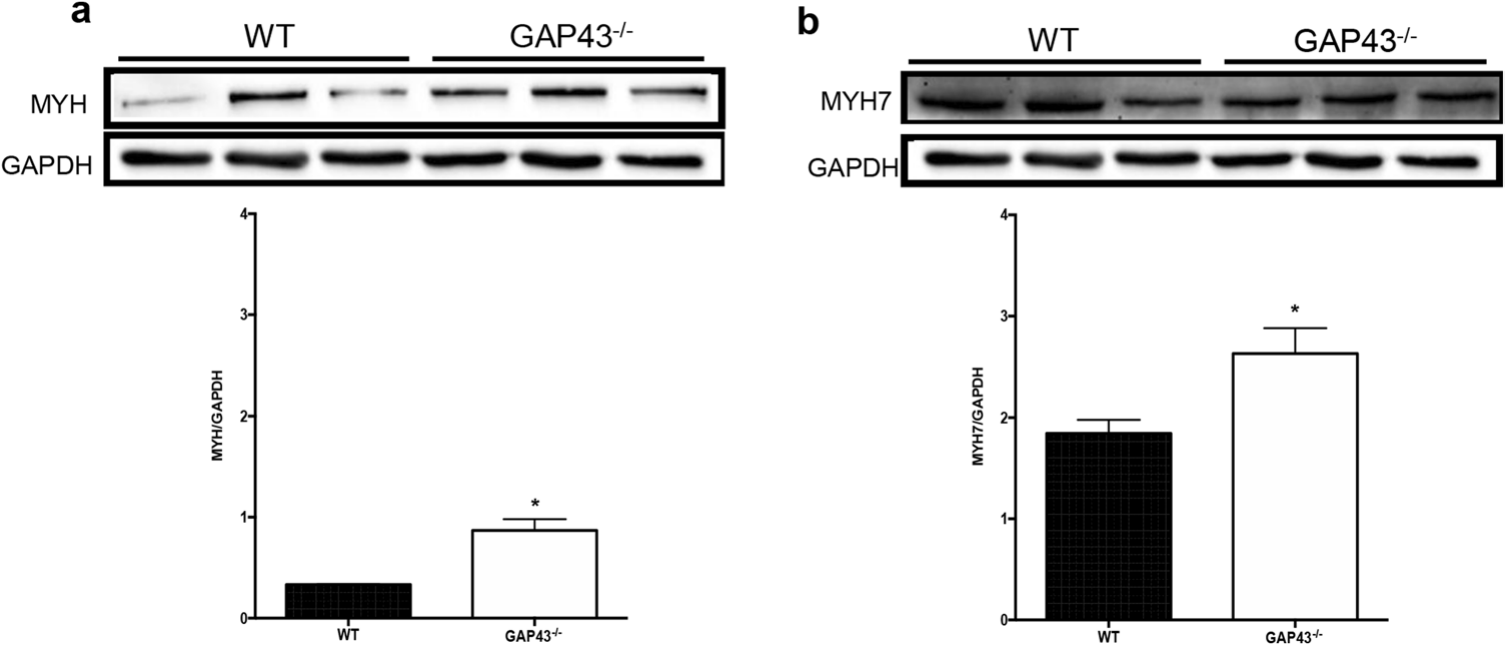
Myosin expression levels. **a** Representative western blot of MYH and the corresponding densitometric analyses in heart samples from WT and GAP43^−/−^ mice; **b** Representative western blot of MYH7 and the corresponding densitometric analyses in heart samples from WT and GAP43^−/−^ mice. The densitometric analyses are plotted as the ratio between the optical density (OD) × mm^2^ of each band and OD0× mm^2^ of the corresponding GAPDH band. Data in in the graphs are expressed as the mean ± SEM from three independent experiments. ^∗^*p* < 0.05 versus WT

The increased heart size found in GAP43^−/−^ mice was further evaluated by quantifying the expression levels of myosin protein and, in particular, the expression levels of total myosin heavy chain (MYH) and its ventricle-specific isoforms (MYH7). Western blot analyses revealed that MYH and MYH7 were both significantly overexpressed in GAP43^−/−^ hearts compared with WT ones (Fig. 4a and b). Comparing the densitometric data, we found that in GAP43^−/−^ hearts the MYH expression was increased by about 2.5-fold (WT 0.3321 ± 0.0032 *N* = 3 versus GAP43^−/−^: 0.8685 ± 0.1122 *N* = 3, *p* = 0.032), while that of ventricular myosin isoform MHY7 increased by about 1.4-fold (WT: 0.8685 ± 0.1122 *N* = 3 versus GAP43^−/−^: 2.635 ± 0.2461 *N* = 3) (Fig. 4a and b).

## Discussion

Our experiments are the first to demonstrate that GAP43 is expressed in heart muscle and that its expression levels are related to the tissue development. Indeed, in neonatal mice, the protein was highly expressed, while its levels decreased when mice reached adult age (3 months). A similar pattern was found in skeletal muscle, where the levels of GAP43 expression were more than doubled in neonatal mice compared with those found in adult (4 months) or elderly (1 year) mice. The decrease in protein expression over time appeared to be related to the maturation of the muscle excitation–contraction structures; thus, due to its peculiar localization near the CRUs, it was proposed that GAP43 is involved in muscle maturation (Caprara et al. 2016). This role could also be played by GAP43 in cardiac development. Indeed, in the early postnatal period, cardiac tissue undergoes a profound rearrangement in the organization of excitation–contraction coupling structures, such as sarcoplasmic reticulum and T tubule development (Piquereau et al. 2010). Also in cardiomyocytes, GAP43 localized in close proximity to α-actinin in a strategic, functional location. Measurements performed in papillary muscle of adult mice showed that the distance between GAP43 spot array was aligned with the sarcomere Z lines in a position close to the T tubule and dyad structures. This localization appeared quite similar to that observed in the I band of mouse skeletal muscle, where the protein appeared closer to the mitochondria than to the Z line (Guarnieri et al. 2013). This dissimilarity could be due to the structural differences between skeletal and cardiac striated muscles, where in the latter there are dyads rather than triads and T tubules form a single strand aligned with Z lines (Kostin et al. 1998; McNutt 1975). Comparing the macroscopic heart features of WT and those of GAP43^−/−^ at 24 h after birth, surprisingly, we observed that the hearts of GAP43^−/−^ mice were significantly heavier than those of WT, despite the body weight of GAP43^−/−^ mice being lower, which was confirmed with the cardiac mass index. In response to hypertrophic signals, cardiomyocytes activate a cellular response characterized by an increase in cell size, without undergoing cell division, and sarcomere assembly that initially compensates for decreased cardiac output (McKinsey and Olson 1999). The degree of hypertrophy was determined by morphometric analysis using hematoxylin–eosin-stained sections. In particular, the measurements of left ventricular wall thickness, septum thickness, and left ventricular free area suggested the presence of hypertrophic growth in homozygous GAP43 knockout mice. The increase in cell size was investigated by confocal microscopy in transversal sections, where the analysis of cross-sectional area confirmed an enlargement of cardiac fibers with increased expression of MYH and the ventricle-specific isoform MYH7 protein. The mammalian heart expresses mainly two myosin heavy chain genes (*MYH6* and *MYH7*), which are the major components of the thick filaments in the sarcomere. These proteins show distinct developmental expression profiles and distributions in the heart. In fact, the MYH7 is the predominant isoform expressed in the ventricular chambers, while MYH6 is preferentially expressed in the atrial chambers (Gordon et al. 2000; Mahdavi et al. 1982). This evidence corroborates our findings in which the increased ventricular thickness and cardiomyocytes cross-sectional area are probably due to increased myofibrillary protein expression. Mechanical overload is the main contributing factor to cardiac hypertrophy (Zou et al. 2004). Depending on the etiopathogenesis, it can be generally differentiated into pressure overload and volume overload, causing morphologically distinct types of cardiac remodeling.

Pressure overload from pathological conditions, such as hypertension and aortic stenosis, occurs during systolic heart work (afterload) and produces concentric hypertrophy. In sharp contrast, volume overload is common in aortic and mitral regurgitation, and occurs during diastole (preload) and yields eccentric hypertrophy (You et al. 2018). Another factor that influences cardiac performance is the mechanical and elastic properties of cardiac tissue, resulting from cellular structure and interaction, as well as from the presence and organization of connective tissue. The development of collagen deposits between cardiac fibers in cardiac fibrosis is another evident change in hearts from GAP43 knockout mice. Considering the tensile strength of collagen and its close association with cardiomyocytes, alterations in interstitial collagen can crucially influence the size and shape of the cardiac chambers as well as ventricular function. Moreover, much evidence in literature has shown that fibrosis increases the diffusion distance of oxygen to cardiomyocytes, negatively affecting metabolic capacity and cardiac function (Toischer et al. 2010).

Many intracellular signaling pathways have been involved in mechanical overload-induced cardiac hypertrophy. These include the MAPK family, including extracellular signal-regulated kinases (ERK1/2), c-Jun NH2-terminal kinase (JNK), and p38 MAPK (He et al. 2016; Jiang et al. 2015; Wu et al. 2014) but also Ca^2+^-related signals such as calcineurin and Ca^2+^/calmodulin-dependent protein kinase II (CaMKII). These also orchestrate cardiac hypertrophy under mechanical overload (van Berlo et al. 2013; Zhou et al. 2010). However, although signaling pathways have been extensively characterized in pressure overload, limited information is available in the case of the involvement in volume overload (You et al. 2018).

GAP43 interacts with CaM in not only neurons but also skeletal muscle. In our previous work, we hypothesized that, by interacting with CaM, GAP43 indirectly modulates the activity of DHPR and RyR channels, and the spontaneous Ca^2+^ oscillations in myotubes. We proposed that the location of GAP43 close to the CRUs in skeletal muscle generates a “functional microdomain” able to anchor CaM near to functional sites like the CRUs (Benowitz and Routtenberg 1997; Caprara et al. 2016).

As reported in other studies, GAP43 expression was not required for embryonic viability, although a high percentage of GAP43^−/−^ mice do not survive beyond weaning (Maier et al. 1999; Metz and Schwab 2004). To date, the reasons for homozygous mice sudden death are not clear. Following autopsy examination, it has been suggested by Strittmatter and colleagues that starvation (stomach and intestine devoid of food) could contribute to death (Strittmatter et al. 1995). Our experiments emphasized that cardiac hypertrophy could be at least a comorbidity factor of sudden death in offspring of GAP-43 knockout mice.

These results pave the way for further studies investigating GAP43 involvement in signaling dynamics at the cellular level.

## Data Availability

All data are available on demand.

## Declarations

### Research involving humans and/or animals

The housing and use of mouse was carried out in accordance with the principles of the Declaration of Helsinki, the Council of the European Community (86/609/EEC), and the Italian government following the regulations for experimental procedures in laboratory animals (92/116).
